# Effect of exercise training with laser phototherapy on homeostasis balance resistant to hypercoagulability in seniors with obesity: a randomized trial

**DOI:** 10.1038/s41598-023-30550-x

**Published:** 2023-03-03

**Authors:** Marwa M. Elsayed, Ghada A. Abdallah, Safaa S. Hassan, Ebtesam N. Nagy

**Affiliations:** 1 Department of Physical Therapy for Cardiovascular/Respiratory Disorder and Geriatrics, Faculty of Physical Therapy, Cairo University, Ahmed El Zyat St., P.O.11432, Dokki, Giza, Egypt; 2 Department of Physical Therapy for Basic Sciences, Faculty of Physical Therapy, Cairo University, Giza, Egypt; 3 Department of Chemistry, Faculty of Science, Cairo University, Giza, Egypt

**Keywords:** Biomarkers, Health care, Health occupations, Medical research, Risk factors

## Abstract

The prevalence of obesity has increased the incidence of obesity-related coagulation disorders. The current study assessed the effectiveness of combined aerobic exercise and laser phototherapy on the coagulation profile and body measurements in older adults with obesity compared to aerobic exercise alone, which has not been adequately explored. We included 76 obese people (50% women and 50% men) with a mean age of 67.83 ± 4.84 years and a body mass index of 34.55 ± 2.67 kg/m^2^. The participants were randomly assigned to the experimental group (which received aerobic training with laser phototherapy) and the control group (which received aerobic training alone) for three months. From the baseline to the final analysis, the absolute changes in specific coagulation biomarker levels (fibrinogen, fibrin fragment D, prothrombin time, Kaolin-Cephalin Coagulation Time), and contributing parameters (C-reactive protein and total cholesterol), were assessed. In comparison to the control group, the experimental group showed significant improvements in all evaluated measures (p < 0.001). So, in comparison to aerobic exercise alone, combined aerobic exercise and laser phototherapy had superior positive effects on coagulation biomarkers and decreased the risk of thromboembolism throughout a three-month intervention period in senior obese persons. Therefore, we suggest adopting laser phototherapy for individuals with a greater risk of hypercoagulability.

The research was entered into the database of clinical trials under the identification NCT04503317.

## Introduction

Obesity is a public health issue coupled with prolonged vascular diseases that get worse by aging^1^, with 28.7 percent is an estimated prevalence between obese Egyptians^2^. Coagulation problems are noticeable in advanced aged-obese individuals who have a high risk of getting thromboembolic events^3^ which is characterized by a high fibrinogen level, fibrin fragment D, and a shorter Kaolin-Cephalin Coagulation Time (KCCT) and prothrombin time (PT)^4^.

Beyond that, hyperlipidemia facilitates the inflammatory cascade, resulting in higher levels of inflammatory mediators like C-reactive protein (CRP) in contrast to healthy people^5^. In people at risk for cardiovascular events, both C-reactive protein and total cholesterol (TC) levels are elevated^6^.

Multimorbidity is prominent among elderly due to the likelihood of adverse consequences being attributed to obesity, hypercoagulability, and hemostasis abnormalities^7^. Exploring other non-pharmacological safety strategies that can influence body weight and achieve a balance in homeostasis is therefore extremely desirable.

Regular exercise training is encouraged by the American College of Sports Medicine as a viable way to minimize the health hazards associated with obesity and the risk of incurring vascular disease^8^, as exercise has a favourable effect on plasma lipid profiles, blood pressure, insulin sensitivity, reduces atherogenesis, and promotes the availability of vasodilators like nitric oxide^9^.

Laser phototherapy is an irradiation approach to the blood that has been used to handle many rheological, neurological, and metabolic cases by enhancing lipoprotein lipase manufacturing, vasodilator release, and microcirculation improvement^10^, additionally reducing the risk of metabolic complications and obesity-related comorbidities^11^ through boosting anti-inflammatory mediators, reduction in fat deposits, and lower serum lipids^12^.

In support of this claim, combining laser phototherapy with other intervention techniques in a variety of disorders maximizes the standalone intervention efficiency results^13^. Although the effect of laser phototherapy has been studied on a variety of medical conditions^14^, no prior studies have evaluated the impact of laser phototherapy alone or in conjunction with other therapies on the coagulation cascade.

So, the purpose of this research was to answer an important clinical question about the efficacy of coupled aerobic exercise and laser phototherapy vs. aerobic exercise on body measures and specific coagulation markers in obese senior individuals.

In our hypothesis, we suggested that combined aerobic exercise and laser phototherapy were more efficient in enhancing the anthropometric measures and certain coagulation biomarkers in comparison to the impact of aerobic exercise separately in senior obese persons.

## Materials and methods

### Study design

This controlled parallel randomized research adopted the latest CONSORT Statement^15^ and was carried out in compliance with the Helsinki Declaration. Prior to the active enrollment of the participants, a written informed consent was gotten from each one after a detailed explanation of the study's objectives and procedures. The study began in September 2020 after getting the institutional ethics board approval of the Faculty of Physical Therapy at Cairo University examined and approved the study's protocol (P.T. REC/012/002724).

### Study setting and participants

The following criteria were included to recruit the participants from Cairo University Hospital's outpatient clinic in Egypt by the research team members: both genders, physically inactive participants (based on the Physical Activity Questionnaire)^16^, between the ages of 60 and 75 years old, and body mass index (BMI) of 30–39.9 kg/m^2^.

While the following were the exclusion criteria: smoking, diabetes, consuming alcohol, uncontrolled hypertensive, cardiac arrythmias, heart failure, participated in a diet program for minimum six months prior to the study, taking blood clotting or body weight-regulating medications, recent illness, cognitive impairment, malignancy diseases, donating blood, or participated in any other research during the 90 days prior to the current study.

Patients with muscle weakness or strain, osteoarthritis, bone fracture, renal and hepatic failure, chest infections, and hyper/hypothyroidism were also eliminated. Under the direction of a qualified physician, the participants were following their prescribed medications (Angiotensin-converting enzyme ACE inhibitors) so long as they didn't affect the findings.

**Figure 1:**
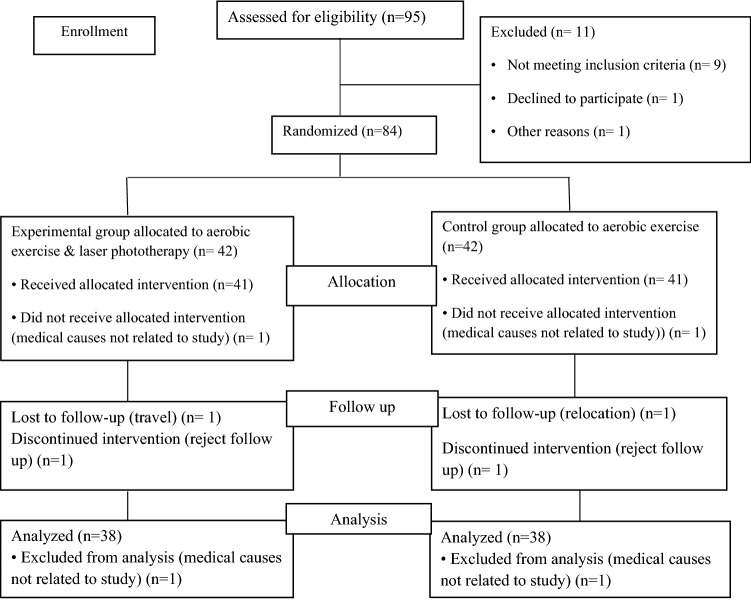
Consort diagram of the present study.

The eligible participants were allocated into two groups equally at random: the experimental group, which engaged in an aerobic training as well as received laser phototherapy through a laser watch, and the control group (performed only the exactly prescribed exercise training program). (Fig. 1).

### Data collection procedure

At screening (at least 5 months before baseline from September 2020 to February 2021), an independent nurse contacted the participants for eligibility by gathering phone call data about their age, health information, and medical history (such as smoking status, comorbidities, and weight loss program participation for the last 6 months).

Study interviews: The eligible participants met in the lab after an overnight fast for at least 12 h at both baseline and post-intervention testing. Demographic data (age, gender, height, weight, waist-hip ratio (WHR), and BMI) and laboratory test data were collected.

The participants had a light breakfast at least two hours before undergoing a submaximal exercise test under the supervision of a well-trained research member (M.M) to determine the proper individualized exercise intensity. Participants returned within a week after all eligibility criteria were confirmed to begin their program. The same protocol was used for the three-month follow-up, which ended for all participants on October 30, 2021, while the entire study was completed on January 30, 2022.

### Randomization

Participants were evenly and randomly assigned to the experimental and control groups. A statistician (not a member of the research team) performed a masked centralized and randomized method with allocation concealment in which the participants and research team members (except the physiotherapists involved in the intervention) were unaware of the assignment. The randomization sequence was created with R Software (version 2.11) and segregated by gender, age (60–75 years), and BMI. To keep an even number of participants in each group, block sizes were varied at random between four and eight. Furthermore, the participants in the control group received their exercise on different days than the participants in the experimental group.

## Intervention

### Aerobic exercise program

Both the experimental and the control groups participated in an aerobic exercise program on separate days using the treadmill KPOWER-Germany, 3 times a week for 12 weeks. Each participant exercised under the supervision of the research physiotherapists and a physician, who monitored the participants' heart rate, blood pressure, and fatigue levels throughout the training. The exercise parameters (intensity and duration) were set in accordance with the recommendations of the American College of Sports Medicine^8^ and the symptom-limited submaximal treadmill exercise test^17^.

The participants exercised at an intensity that ranged from 60% of the maximum heart rate in the first six weeks to 75% of the maximum heart rate in the last six weeks^8^, as the exercise session consisted of three phases over 60 min (warming up and cooling down phases for 10 min at 40% of the maximum heart rate, and in between phases according to the pre-prescribed exercise intensity as mentioned for 40 min). All sessions were conducted at the Faculty of Physical Therapy-outpatient clinic in Egypt, with good access to equipment.

### Laser phototherapy parameters

Coherence infra-red laser treatment (650 nm) was administered to individuals in the experimental group only using a laser wristwatch (LASPOT, Wuhan, China). The laser watch stimulated specific wrist acupuncture points while simultaneously releasing two nasal laser beams using two nasal probes (radiated both ulnar and radial arteries, the middle wrist acupoints)^10^ There were no unfavorable therapy reviews reported.

The Laser Watch's operational parameters were monochromatic 650 nm wavelength, energy (8.64 J), spot size (0.03 cm^2^), peak power (5 mW), energy density (288 J/cm^2^), frequency (3 sessions per week for 12 weeks), continuous emission mode, and GaAIAs was the emission medium. Prior to each application, the equipment's power was calibrated; the power measurement density (w/cm^2^) was 0.16; and the session lasted for 60 min.

### Primary outcomes

#### Blood analysis

Samples of venous blood were obtained by an independent nurse from each participant after fasting for 12–14 h at 8–9 AM and frozen at − 80 °C until the analysis to evaluate fibrinogen, KCCT, PT, serum TC, fibrin fragment D, and CRP levels. INNOVANCE® fibrin fragment D (Siemens Healthcare Diagnostic Products GmbH) was used to measure plasma fibrin fragment D levels by a particle-enhanced immunoturbidimetric assay for quantifying cross-linked fibrin degradation products (fibrin fragment D). All samples were analyzed by the same accredited medical laboratory before the intervention and after its completion^18^.

### Secondary outcomes

#### Body measures

- BMI was measured automatically using a standard scale (TR-200 LP-China with a precision of ± 0.1 kg and 0.1 cm and a capacity of 200 kg) depending on this formula (kg/m^2^) by recording the volunteers' bodyweights (kg) and lengths (m^2^)^19^.
- WHR: It was measured by dividing the participants’ circumferences of their waists and hips. In the standing position, the hip circumference is equal to the circumference around the bilateral greater trochanters, while the waist was measured at the place where the iliac crest and lower rib met (in cm)^20^.

### Statistical analysis

#### Sample size

For sample size calculation, G* power statistical software (version 3. 1. 9. 2; Franz Faul, Universitat Kiel, Germany) was used based on the primary outcome (fibrinogen). The power calculation was done as a comparison between two groups (experimental versus control) according to the independent t-test to gain 80% power, 95% confidence interval, alpha = 0.05, and an effect size of 0.537 was obtained from a 20-subject pilot study. To allow for an expected 10% dropout, the appropriate required sample size for the study was n = 76.

Shapiro–Wilk test data distribution was determined using statistical analysis software (SPSS version 25, Armonk, NY: IBM Corp), and continuous data were expressed using mean ± standard deviation (SD). Both dependent and independent sample t-tests were utilized to compare variables within or between experimental and control groups for comparison before and after the study was completed (with statistical significance for *p*-values < 0.05). We used the minimally clinically important difference (MCID) of coagulation outcomes to assess if the study's effect is important to patients. The MCID estimate depends on comparing the mean difference (MD) in pre- and post-intervention of coagulation factors between the two groups.

## Results

### Participants’ characteristics

There were 76 participants (50 percent women and 50 percent men), with an average age and weight of 67.83 ± 4.84 years and 84.97 ± 3.93 kg, respectively, and a body mass index of 34.55 ± 2.67 kg/m^2^. The experimental group (38 patients) underwent laser phototherapy along with aerobic exercise, while the control group (38 patients) underwent aerobic exercise alone.

Age, weight, height, BMI, WHR, and determined serum coagulation markers (fibrinogen, fibrin fragment D, prothrombin time, KCCT), and contributing parameters (C-reactive protein, and TC) showed no statistically significant differences between the two groups' baseline characteristics (Table 1) (*p* > 0.05).

**Table 1:** Baseline characteristics of the study participants.

Characteristics			Experimental group (n = 19 ♂ &19 ♀)	Control group (n = 19 ♂ &19 ♀)	*p*-value
Age (yrs)		Mean ± SD	68.31 ± 5.17	67.35 ± 4.50	0.953
		Range	60.00–74.80	60.00–74.70	
Height (cm)		Mean ± SD	155.39 ± 4.13	156.82 ± 4.38	0.276
		Range	148.00–165.00	149.00–166.00	
Weight (kg)		Mean ± SD	85.41 ± 3.63	84.53 ± 4.23	0.093
		Range	78.52–92.82	77.55–91.87	
BMI (kg/m^2^)		Mean ± SD	34.63 ± 2.81	34.47 ± 2.53	0.721
		Range	30.50–39.60	30.60–39.50	
WHR	Men	Mean ± SD	0.91 ± 1.23	0.92 ± 0.33	0.626
		Range	0.83–0.86	0.81–0.81	
	women	Mean ± SD	0.81 ± 0.27	0.82 ± 0.44	0.551
		Range	0.77–0.82	0.78–0.16	
PT (s)		Mean ± SD	11.71 ± 0.76	11.54 ± 0.68	0.832
		Range	10.30–12.70	10.40–12.60	
KCCT (s)		Mean ± SD	27.75 ± 1.21	27.60 ± 1.14	0.591
		Range	26.30–29.60	26.00–29.50	
Fibrinogen (g/l)		Mean ± SD	3.87 ± 0.42	3.76 ± 0.52	0.720
		Range	3.34–4.56	3.25–4.38	
T.C (mg/dl)		Mean ± SD	212.83 ± 13.17	213.54 ± 13.80	0.726
		Range	187.00–238.00	189.00–236.00	
CRP (mg/l)		Mean ± SD	4.56 ± 0.42	4.35 ± 0.60	0.574
		Range	3.33–5.16	3.11–5.15	
fibrin fragment D (ng/mL)		Mean ± SD	224.67 ± 30.80	223.58 ± 29.45	0.610
		Range	181.00–278.00	180.00–277.00	

BMI, Body mass index; CRP, c—reactive protein; KCCT, Kaolin Cephalin Coagulation Time; PT, partial thrombin; TC, Total cholesterol; WHR, Waist-hip ratio; ♂, men; ♀,women. Data represented as mean ± standard deviation (SD).

### Primary outcomes of coagulation factors

According to a paired sample t-test, Table 2 demonstrates statistically significant differences in the primary outcomes of coagulation factors between the experimental group and the control group before and after the intervention (*p* < 0.001) compared to the control group (*p* < 0.01), with a more significant decline in the experimental group than in the control group following study completion (*p* < 0.001) according to an independent sample t-test. Compared to the control group, the experimental group had a statistically significant improvement from pre- to post-intervention in their PT (MD = 1.65 s vs. 0.64 s; p < 0.001) and KCCT (4.46 s vs. 1.76 s; p < 0.001). However, the experimental group had a more significant decrease from baseline to after intervention in Fibrinogen (MD = −0.94 g/l), TC (MD = −37.35 mg/dl), CRP (MD = −1.56 mg/l), and fibrin fragment D (MD = −33.51 ng/mL) compared to the control group (MD = −0.31 g/l, −10.81 mg/dl, −0.33 mg/l and −11.03 ng/mL, respectively) (for all p < 0.001 according to an independent sample t-test).

**Table 2:** Comparison of primary outcomes of coagulation factors before and after 3 months of intervention between both groups.

Variables	Experimental group (n = 19 ♂ &19 ♀)		P_aired t-test_ -	MD	Control group (n = 19 ♂ &19 ♀)		P_aired t-test_ -	MD	P_Indep.t-test_
	Pre	Post	Value		Pre	Post	Value		value
PT (s)	11.71 ± 0.76	13.36 ± 0.89	< 0.001*	1.65	11.54 ± 0.68	12.18 ± 0.45	< 0.01*	0.64	< 0.001**
KCCT (s)	27.75 ± 1.21	32.21 ± 2.13	< 0.001*	4.46	27.60 ± 1.14	29.36 ± 2.15	< 0.01*	1.76	< 0.001**
Fibrinogen (g/l)	3.87 ± 0.42	2.93 ± 1.04	< 0.001*	−0.94	3.76 ± 0.52	3.45 ± 2.42	< 0.01*	−0.31	< 0.001**
TC (mg/dl)	212.83 ± 13.17	175.48 ± 13.27	< 0.001*	−37.35	213.54 ± 13.80	202.73 ± 13.02	< 0.01*	−10.81	< 0.001**
CRP (mg/l)	4.56 ± 0.42	3.00 ± 0.31	< 0.001*	−1.56	4.35 ± 0.60	4.02 ± 0.61	< 0.01*	−0.33	< 0.001**
fibrin fragment D (ng/mL)	224.67 ± 30.80	191.16 ± 24.74	< 0.001*	−33.51	223.58 ± 29.45	212.55 ± 24.80	< 0.01*	−11.03	< 0.001**

CRP, c—reactive protein; KCCT, Kaolin Cephalin Coagulation Time; Total cholesterol, TC; ♂, men; ♀,women.

Data represented as mean ± standard deviation (SD).

*Statistically significant at *p* < 0.05 according to paired sample t-test.

**Statistically significant at *p* < 0.05 according to independent sample t-test.

### Secondary outcomes of anthropometric measures

Table 3 shows statistically significant differences in anthropometric measures between the pre and post-intervention in the experimental group (*p* < 0.001) compared to the control group (*p* < 0.01) according to a paired sample t-test, with a more significant reduction in the experimental group than in the control group after the study's completion (*p* < 0.001) according to an independent sample t-test.

**Table 3:** Comparison of secondary outcome of body measures before and after 3 months of intervention between both groups.

Variables		Experimental group (n = 19 ♂ &19 ♀)		P_aired t-test_ -	Control group (n = 19 ♂ &19 ♀)		P_aired t-test_ -	P_Indep.t-test_
		Pre	Post	Value	Pre	Post	Value	value
Weight (kg)		85.41 ± 3.63	74.23 ± 4.02	< 0.001*	84.53 ± 4.23	79.40 ± 5.27	< 0.01*	< 0.001**
BMI (kg/m^2^)		34.63 ± 2.81	30.20 ± 3.11	< 0.001*	34.47 ± 2.53	32.29 ± 2.78	< 0.01*	< 0.001**
WHR	♂	0.91 ± 1.23	0.85 ± 1.03	< 0.001*	0.92 ± 0.33	0.90 ± 1.11	< 0.01*	< 0.001**
	♀	0.81 ± 0.27	0.75 ± 0.12	< 0.001*	0.82 ± 0.44	0.80 ± 0.37	< 0.01*	< 0.001**

BMI, Body mass index; CRP, C-reactive protein; WHR, Waist-hip ratio; ♂, men; ♀, women.

Data represented as mean ± standard deviation (SD).

*Statistically significant at *p* < 0.05 according to paired sample t-test.

**Statistically significant at *p* < 0.05 according to independent sample t-test.

### Adverse events of applied intervention

Over the course of this study, no adverse events from laser phototherapy application or aerobic exercise were reported, as documented in weekly interviews to record any adverse events experienced by the participants.

## Discussion

Numerous studies have demonstrated the positive effects of aerobic exercise and laser phototherapy on people with a variety of health issues^11–13^, particularly highly risk senior individuals who lead sedentary life^21^.

The findings revealed a significant decrease (*p* < 0.001) in the participants' body measurements (WHR, weight, and BMI). This was in line with the results of Marandi et al^22^, who discovered a significant decline in these measurements in obese/overweight females following aerobic training for 14 weeks (*p* < 0.001). As well as Caruso-Davis et al^23^ who discovered a significant reduction in body measures (*p* < 0.05) after laser application for 30 min twice a week for four weeks with both genders.

The mechanism by which aerobic exercise decreases an individual's body weight is by using deposed fat as a fuel^24^. Whereas the laser phototherapy breaks the lipid, which is quickly eliminated through the lymphatic and re-esterified in other tissues or metabolized for energy^25^. According to Da Silveira Campos et al^26^, the combination of aerobic exercise and laser phototherapy enhanced outcomes on markers of fat tissue trans-differentiation by raising body energy expenditure and potentially promoting body mass loss, resulting in improved control of obesity and associated comorbidities^27^.

Additionally, in this study, TC was significantly lower in the experimental group compared with the control group (*p* < 0.001), consistent with Ngayimbesha et al.'s finding^28^ that TC dramatically decreased following eight weeks of aerobic activity in obese people. Exercise appears to improve skeletal muscle's capacity to use lipids rather than glycogen, which led to lowering plasma cholesterol levels. Additionally, according to LIU^29^, for 30 min each time once a day ten days of laser phototherapy led to significant lower of TC (*p* < 0.05) in patients with coronary heart disease through increasing the release of lipoprotein lipase and endorphin^10^. As a result, combining laser phototherapy with other treatment measures amounted to a greater effect on body weight and cholesterol level, in line with Mekawy et al.'s findings^27^.

This study's findings show a more significant drop in CRP in the experimental group compared to the control group (*p* < 0.001). In line with this, Arikawa et al^30^ demonstrated that a 16-wk aerobic exercise program significantly decreased levels of CRP in young women, especially in those who were obese (*p* = 0.040). Elsayed et al^31^ demonstrated that CRP significantly decreases after 3 months of laser biostimulation and diet in obese postmenopausal women.

It is suggested that both aerobic exercise and laser phototherapy have anti-inflammatory benefits by increasing the amount of nitric oxide released from endothelial tissue^32^ and lowering the production of inflammatory cytokines from the smooth muscles of the endothelium tract. Both of these improve the lipolysis process, which reduces the body fat proportion^27^ which is the primary sources of inflammatory cytokines like interleukin 6, which cause the liver to release CRP^33^.

We also noted that the addition of laser phototherapy to aerobic exercise resulted in greater fibrinolysis, with a substantial decrease in fibrinogen, fibrin fragment D, and an increase in PT and KCCT (*p* < 0.001) compared to aerobic training alone (*p* < 0.01). Our findings correspond with those of Khalil et al^34^, who showed that aerobic exercise had an anti-coagulable impact by increasing the time needed for blood to clot and lowering serum fibrinogen.

After moderate to vigorous aerobic exercise, Parsons et al^35^ likewise observed after 10 sessions of laser phototherapy a reduction in the fibrin fragment D concentration (*p* ≤ 0.006). Additionally, increase time of KCCT and PT in patients with myocardial infarction after 10 sessions of laser phototherapy according to Volov et al^36^.

Aerobic exercise and laser phototherapy enhance fibrinolysis activity by reducing body fat, which is considered the primary activator of hepatic fibrinogen, which is favorably correlated with fibrin fragment D^11^. Besides that, aerobic exercise lowers levels of fibrinogen by raising catecholamines and neurotransmitters like adrenaline, norepinephrine, and epinephrine^37^. Additionally, laser phototherapy and aerobic activity increase nitric oxide release^38^, trigger endothelial cells to release tissue plasminogen activator and reduce the synthesis of plasminogen activator inhibitor 1 platelet^39^.

The results of this study highlighted the effect of aerobic exercise and laser phototherapy on the risk of thromboembolism in aged obese people which in same line with van Stralen et al^11, 40^.

### Strengths and limitations of the study

The study focused on senior obese people who are at a considerable risk of acquiring life-threatening vascular diseases. These results indicate a preventive strategy to lower certain coagulation indicators in this population that combines aerobic exercise with laser phototherapy. Our findings suggest that high risk individuals may benefit from laser phototherapy to minimize coagulation and complications.

Despite the favourable results of this study, there are several restrictions that need to be considered. The results may only be generalizable to other populations depending on the topic selection criteria, to start. We only used one exercise program and one laser phototherapy wavelength, so we encouraged further studies with longer duration and with follow up to assess the impact of various exercise regimens and intensities or different laser phototherapy wavelengths on other coagulation biomarkers.

## Conclusion

The results of this study provide evidence that laser phototherapy in combination with aerobic training is a practical and affordable technique to enhance the health status of senior obese adults by enhancing anthropometric and particular coagulation indicators and lowering the risk of thromboembolism. Finally, we advise further research about the effects of different laser wave lengths and exercise intensities, especially on highly risky people who can’t perform vigorous exercise.

## Data Availability

The current study analyzed data will be available on a reasonable request from the corresponding author.
